# (*R*)-2-Phen­oxy-1-(4-phenyl-2-sulfan­ylidene-1,3-oxazolidin-3-yl)ethanone

**DOI:** 10.1107/S160053681103858X

**Published:** 2011-09-30

**Authors:** Ignez Caracelli, Daniel C. S. Coelho, Paulo R. Olivato, Thiago C. Correra, Alessandro Rodrigues, Edward R. T. Tiekink

**Affiliations:** a BioMat-Departamento de Física, Universidade Federal de São Carlos, CP 676, 13565-905, São Carlos, SP, Brazil; b Laboratório de Cristalografia, Estereodinâmica e Modelagem Molecular, Universidade Federal de São Carlos, Departamento de Química, CP 676, 13565-905, São Carlos, SP, Brazil; c Chemistry Institute, Universidade de São Paulo, 05508-000 São Paulo, SP, Brazil; d Departamento de Ciencias Exatas e da Terra, Universidade Federal de São Paulo, UNIFESP, Diadema, Brazil; e Department of Chemistry, University of Malaya, 50603 Kuala Lumpur, Malaysia

## Abstract

The central 1,3-oxazolidine-2-thione ring in the title compound, C_17_H_15_NO_3_S, is approximately planar with maximum deviations of 0.036 (4) and −0.041 (5) Å for the O and methyl­ene-C atoms, respectively. The dihedral angles formed between this plane and the two benzene rings, which lie to the same side of the central plane, are 86.5 (2) [ring-bound benzene] and 50.6 (3)°. The ethan-1-one residue is also twisted out of the central plane, forming a O—C—N—C torsion angle of 151.5 (5)°. The dihedral angle formed by the benzene rings is 62.8 (2)° so that overall, the mol­ecule has a twisted U-shape. In the crystal, mol­ecules are linked into supra­molecular arrays two mol­ecules thick in the *bc* plane through C—H⋯O, C—H⋯S and C—H⋯π inter­actions.

## Related literature

For background to oxazolidine-2-thio­nes, see: Evans *et al.* (1981 ▶); Crimmins & King (1998 ▶); Zhang *et al.* (2004) ▶; Shinisha & Sunoj (2010 ▶); Tamura *et al.* (2009 ▶). For related structures, see: Kitoh *et al.* (2002 ▶). For the synthesis, see: Wu *et al.* (2004 ▶); Rodrigues *et al.* (2005 ▶).
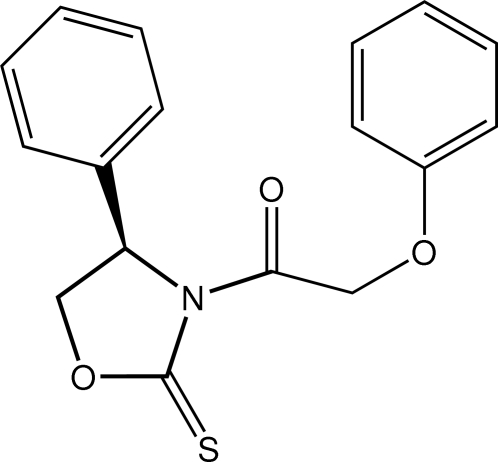


## Experimental

### 

#### Crystal data

C_17_H_15_NO_3_S
*M* *_r_* = 313.37
Monoclinic, 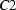

*a* = 33.514 (3) Å
*b* = 5.7514 (6) Å
*c* = 7.7172 (8) Å
β = 93.808 (7)°
*V* = 1484.2 (3) Å^3^
*Z* = 4
Mo *K*α radiation
μ = 0.23 mm^−1^
*T* = 126 K
0.30 × 0.25 × 0.16 mm

#### Data collection

Bruker APEXII CCD diffractometer
7408 measured reflections
2588 independent reflections
2166 reflections with *I* > 2σ(*I*)
*R*_int_ = 0.064

#### Refinement

*R*[*F* ^2^ > 2σ(*F* ^2^)] = 0.066
*wR*(*F* ^2^) = 0.180
*S* = 1.08
2588 reflections
199 parameters
1 restraint
H-atom parameters constrained
Δρ_max_ = 0.81 e Å^−3^
Δρ_min_ = −0.47 e Å^−3^
Absolute structure: Flack (1983 ▶), 1143 Friedel pairs
Flack parameter: 0.01 (18)

### 

Data collection: *APEX2* (Bruker, 2007 ▶); cell refinement: *SAINT* (Bruker, 2007 ▶); data reduction: *SAINT*; program(s) used to solve structure: *SIR97* (Altomare *et al.*, 1999 ▶); program(s) used to refine structure: *SHELXL97* (Sheldrick, 2008 ▶); molecular graphics: *ORTEP-3* (Farrugia, 1997 ▶) and *DIAMOND* (Brandenburg, 2006 ▶); software used to prepare material for publication: *MarvinSketch* (Chemaxon, 2010 ▶) and *publCIF* (Westrip, 2010 ▶).

## Supplementary Material

Crystal structure: contains datablock(s) global, I. DOI: 10.1107/S160053681103858X/hg5095sup1.cif

Structure factors: contains datablock(s) I. DOI: 10.1107/S160053681103858X/hg5095Isup2.hkl

Supplementary material file. DOI: 10.1107/S160053681103858X/hg5095Isup3.cml

Additional supplementary materials:  crystallographic information; 3D view; checkCIF report

## Figures and Tables

**Table 1:** Hydrogen-bond geometry (Å, °) *Cg*1 and *Cg*2 are the centroids of the C4–C9 and C12–C17 rings, respectively.

*D*—H⋯*A*	*D*—H	H⋯*A*	*D*⋯*A*	*D*—H⋯*A*
C5—H5⋯O2^i^	0.95	2.29	3.202 (6)	162
C9—H9⋯O3^ii^	0.95	2.56	3.350 (6)	140
C11—H11b⋯S^iii^	0.99	2.87	3.814 (5)	160
C17—H17⋯*Cg*1^iv^	0.95	2.99	3.703 (5)	133
C8—H8⋯*Cg*2^ii^	0.95	2.79	3.523 (6)	135

Symmetry codes: (i) 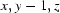; (ii) 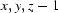; (iii) 
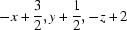
; (iv) 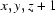.

**Fig. 1.**
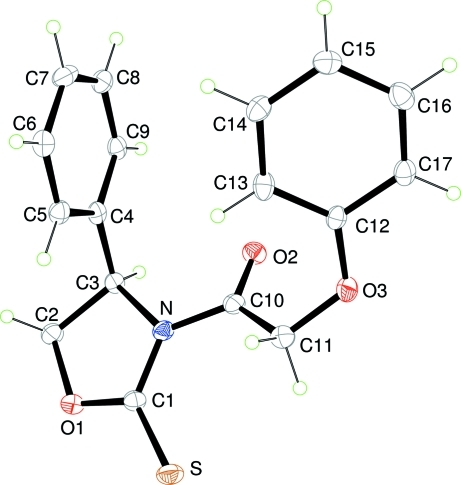
The molecular structure of (I) showing atom labelling scheme and displacement ellipsoids at the 50% probability level (arbitrary spheres for the H atoms).

**Fig. 2.**
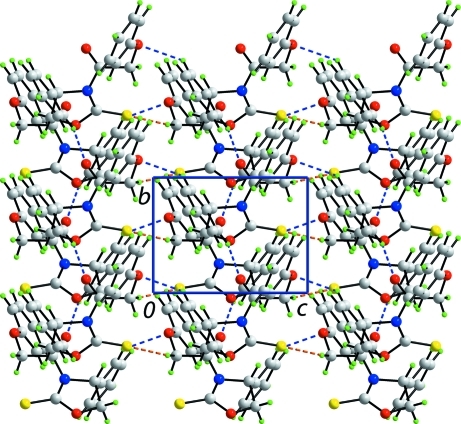
A view in projection of the supramolecular 2-D array with a thickness corresponding to two molecules. The C—H···O and C—H···S contacts are shown as blue and orange dashed lines, respectively.

**Fig. 3.**
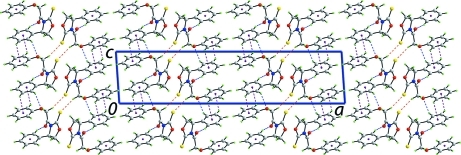
A view in projection down the b axis of the unit-cell contents of (I) highlighting the stacking of layers along the a axis. The C—H···O, C—H···S and C—H···π contacts are shown as blue, orange and purple dashed lines, respectively.

## supplementary crystallographic information

### Comment

Since the first report in 1981 (Evans *et al.* 1981) exploring oxazolidin-2-ones as chiral auxiliaries in enantioselective aldol condensations, a number of related oxazolidin-2-ones and their synthetic applications have been reported. Recently, studies have shown that sulfur oxazolidine-2-thione derivatives have some advantages in terms of the asymmetric induction over the original 2-oxo analogues (Crimmins *et al.*, 1998; Zhang *et al.*, 2004; Shinisha *et al.*, 2010). For this reason, the use of oxazolidine-2-thiones as chiral auxiliaries is a widely employed strategy for the total synthesis of relevant biological compounds (Tamura *et al.*, 2009). An interesting study reported by Kitoh and collaborators (Kitoh *et al.*, 2002) showed an alternative route to chiral 4-phenyl-1,3-oxazolidine-2-thione by optical resolution of the racemate through preferential crystallization. In this study, the crystal structure and vibrational spectra analysis of chiral 4-phenyl-1,3-oxazolidine-2-thione, (I), are reported.

The molecular structure of (I), Fig. 1, features a planar 1,3-oxazolidine-2-thione ring with the maximum deviations from the least-squares plane being 0.036 (4) for atom O1 and -0.041 (5) for atom C2. With reference to this plane, the two benzene rings are orientated to the same side and form dihedral angles of 86.5 (2) [ring-bound benzene ring] and 50.6 (2) °, respectively, with it. The ethan-1-one group is not co-planar with the five-membered ring as seen in the value of the O2—C10—N—C1 torsion angle of 151.5 (5) °; the carbonyl-O2 atom lies to the opposide side of the central plane to the benzene rings. The dihedral angle formed between the two benzene rings of 62.8 (2) ° indicates a non-parallel alignment. Overall, the molecule of (I) adopts a twisted U-shape.

The presence of C—H···O and C—H···S contacts, Table 1, leads to the formation of supramolecular 2-D arrays in the *bc*-plane, Fig. 2. The layers are two molecules thick. Additional stability to the layers is afforded by C—H···π interactions, Table 1. The layers with a flat topology stack along the *a*-direction, Fig. 3.

### Experimental

The starting *(R)*-4-phenyloxazolidine-2-thione was synthesized from *(R)*-phenylglycine in three steps as previously reported (Wu *et al.* 2004). The phenoxyacetyl-oxazolidine- 2-thione derivative was prepared by acylation of *(R)*-4- phenyloxazolidine-2-thione (Rodrigues *et al.* 2005). The title compound was then obtained by adding DCC (*N,N'*-dicyclohexylcarbodiimide) (690 mg, 3.35 mmol) in one portion to an ice-cooled solution of *(R)*-4-phenyloxazolidine-2-thione (500 mg, 2.79 mmol), *N,N*-dimethylpyridin-4-amine (34 mg, 0.28 mmol) and 2-phenoxyacetic acid (510 mg, 3.35 mmol) in methylene chloride (10 ml). The resulting suspension, kept under a nitrogen atmosphere during the reaction time, was then allowed to reach r.t. After 48 h under stirring, the dicyclohexylurea formed was filtered off and the precipitate washed with methylene chloride (10 ml). The organic layers were washed with a sat. aq. solution of NaHCO_3_ (20 ml) and dried over Na_2_SO_4_. Filtration and evaporation of the solvent in vacuum gave the crude product which was purified by flash column chromatography on silica gel with 30% acetone in hexanes to give the pure product as a white solid (320 mg, 37%). Colourless crystals of the compound were obtained by vapour diffusion from hexane/acetone at 298 K: m.p. = 385–387 K;[α]_D_^25^ -66.7° (*c* 1.8, CHCl_3_); ^1^H NMR (300 MHz, CDCl_3_/TMS), δ (p.p.m.): 7.40–7.29 (m, 5H), 7.26–7.21 (m, 2H), 6.98–6.92 (m, 1H), 6.85–6.82 (m, 2H), 5.71 (dd,^3^*J* = 8.65 Hz, ^3^*J* = 3.24 Hz, 1H), 5.55 (AB system,Δν = 56.0 Hz, ^2^*J* = 17.7 Hz, 1H). ^3^C NMR (75 MHz, CDCl_3_/TMS); δ(p.p.m.): 184.93, 168.69, 157.60, 137.99, 129.52, 129.32, 129.14, 126.29, 121.70, 114.76, 75.26, 69.34, 62.07. Anal. calcd. for C_17_H_15_NO_3_S: C,65.16%; H,4.82%, N, 4.47%. Found: C,64.48%; H,4.71%, N, 4.18%. Mass Spectra: *M*+ = 314.0836, Exact mass = 314.0845.

### Refinement

The H atoms were geometrically placed (C–H = 0.95–1.00 Å) and refined as riding with *U_iso_*(H) = 1.2*U_eq_*(C).

### Crystal data

**Table:**

C_17_H_15_NO_3_S	*F*(000) = 656
*M**_r_* = 313.37	*D*_x_ = 1.402 Mg m^−^^3^
Monoclinic, *C*2	Mo *K*α radiation, λ = 0.71073 Å
Hall symbol: C 2y	Cell parameters from 2062 reflections
*a* = 33.514 (3) Å	θ = 2.7–26.4°
*b* = 5.7514 (6) Å	µ = 0.23 mm^−^^1^
*c* = 7.7172 (8) Å	*T* = 126 K
β = 93.808 (7)°	Block, colourless
*V* = 1484.2 (3) Å^3^	0.30 × 0.25 × 0.16 mm
*Z* = 4	

### Data collection

**Table:**

Bruker APEXII CCD diffractometer	2166 reflections with *I* > 2σ(*I*)
Radiation source: fine-focus sealed tube	*R*_int_ = 0.064
graphite	θ_max_ = 25.0°, θ_min_ = 2.7°
φ and ω scans	*h* = −39→36
7408 measured reflections	*k* = −6→6
2588 independent reflections	*l* = −8→9

### Refinement

**Table:**

Refinement on *F*^2^	Secondary atom site location: difference Fourier map
Least-squares matrix: full	Hydrogen site location: inferred from neighbouring sites
*R*[*F*^2^ > 2σ(*F*^2^)] = 0.066	H-atom parameters constrained
*wR*(*F*^2^) = 0.180	*w* = 1/[σ^2^(*F*_o_^2^) + (0.1039*P*)^2^ + 1.9443*P*] where *P* = (*F*_o_^2^ + 2*F*_c_^2^)/3
*S* = 1.08	(Δ/σ)_max_ < 0.001
2588 reflections	Δρ_max_ = 0.81 e Å^−^^3^
199 parameters	Δρ_min_ = −0.47 e Å^−^^3^
1 restraint	Absolute structure: Flack (1983), 1143 Friedel pairs
Primary atom site location: structure-invariant direct methods	Flack parameter: 0.01 (18)

### Special details

**Table:**

Geometry. All s.u.'s (except the s.u. in the dihedral angle between two l.s. planes) are estimated using the full covariance matrix. The cell s.u.'s are taken into account individually in the estimation of s.u.'s in distances, angles and torsion angles; correlations between s.u.'s in cell parameters are only used when they are defined by crystal symmetry. An approximate (isotropic) treatment of cell s.u.'s is used for estimating s.u.'s involving l.s. planes.
Refinement. Refinement of *F*^2^ against ALL reflections. The weighted *R*-factor *wR* and goodness of fit *S* are based on *F*^2^, conventional *R*-factors *R* are based on *F*, with *F* set to zero for negative *F*^2^. The threshold expression of *F*^2^ > 2σ(*F*^2^) is used only for calculating *R*-factors(gt) *etc*. and is not relevant to the choice of reflections for refinement. *R*-factors based on *F*^2^ are statistically about twice as large as those based on *F*, and *R*- factors based on ALL data will be even larger.

### Fractional atomic coordinates and isotropic or equivalent isotropic displacement parameters (Å^2^)

**Table:**

	*x*	*y*	*z*	*U*_iso_*/*U*_eq_	
C1	0.71650 (13)	0.0824 (8)	0.6314 (6)	0.0265 (11)	
C2	0.70382 (14)	0.0160 (10)	0.3371 (6)	0.0298 (11)	
H2A	0.6857	−0.1125	0.2984	0.036*	
H2B	0.7219	0.0499	0.2439	0.036*	
C3	0.67974 (14)	0.2332 (8)	0.3797 (6)	0.0248 (10)	
H3	0.6906	0.3717	0.3202	0.030*	
C4	0.63525 (14)	0.2206 (8)	0.3422 (6)	0.0240 (10)	
C5	0.61306 (13)	0.0403 (10)	0.4132 (6)	0.0284 (10)	
H5	0.6263	−0.0765	0.4823	0.034*	
C6	0.57230 (14)	0.0333 (10)	0.3826 (6)	0.0311 (10)	
H6	0.5574	−0.0873	0.4323	0.037*	
C7	0.55245 (14)	0.2019 (10)	0.2792 (7)	0.0332 (12)	
H7	0.5243	0.1948	0.2564	0.040*	
C8	0.57425 (15)	0.3789 (9)	0.2104 (7)	0.0311 (11)	
H8	0.5609	0.4950	0.1409	0.037*	
C9	0.61494 (15)	0.3893 (8)	0.2410 (6)	0.0274 (11)	
H9	0.6295	0.5126	0.1928	0.033*	
C10	0.67735 (13)	0.4532 (9)	0.6533 (7)	0.0272 (11)	
C11	0.67426 (15)	0.4547 (9)	0.8496 (7)	0.0318 (12)	
H11A	0.6608	0.3109	0.8856	0.038*	
H11B	0.7014	0.4586	0.9086	0.038*	
C12	0.61226 (14)	0.6591 (8)	0.8538 (6)	0.0242 (10)	
C13	0.59105 (15)	0.4913 (9)	0.7557 (6)	0.0310 (12)	
H13	0.6045	0.3586	0.7149	0.037*	
C14	0.55026 (14)	0.5183 (10)	0.7176 (6)	0.0319 (11)	
H14	0.5360	0.4038	0.6498	0.038*	
C15	0.52995 (15)	0.7094 (10)	0.7768 (7)	0.0343 (12)	
H15	0.5019	0.7247	0.7525	0.041*	
C16	0.55166 (17)	0.8801 (10)	0.8735 (7)	0.0358 (12)	
H16	0.5382	1.0130	0.9138	0.043*	
C17	0.59217 (15)	0.8566 (9)	0.9103 (6)	0.0291 (11)	
H17	0.6066	0.9742	0.9741	0.035*	
N	0.68964 (11)	0.2510 (7)	0.5738 (5)	0.0255 (9)	
O1	0.72682 (10)	−0.0467 (6)	0.4973 (4)	0.0305 (8)	
O2	0.66648 (10)	0.6161 (6)	0.5650 (4)	0.0300 (8)	
O3	0.65227 (10)	0.6512 (6)	0.8994 (4)	0.0306 (8)	
S	0.73540 (4)	0.0379 (2)	0.82903 (16)	0.0340 (4)	

### Atomic displacement parameters (Å^2^)

**Table:**

	*U*^11^	*U*^22^	*U*^33^	*U*^12^	*U*^13^	*U*^23^
C1	0.023 (2)	0.030 (3)	0.026 (3)	0.0007 (18)	0.0016 (18)	0.001 (2)
C2	0.035 (2)	0.034 (3)	0.021 (2)	0.003 (2)	−0.0009 (18)	0.000 (2)
C3	0.037 (3)	0.025 (2)	0.012 (2)	0.004 (2)	0.0019 (18)	0.0023 (19)
C4	0.035 (3)	0.023 (2)	0.014 (2)	0.0003 (19)	0.0044 (18)	−0.0011 (19)
C5	0.035 (2)	0.026 (2)	0.024 (2)	−0.002 (2)	−0.0004 (18)	0.001 (2)
C6	0.037 (3)	0.028 (2)	0.029 (3)	−0.003 (2)	0.0054 (19)	−0.002 (3)
C7	0.023 (2)	0.049 (3)	0.027 (3)	0.000 (2)	−0.002 (2)	−0.006 (2)
C8	0.037 (3)	0.035 (3)	0.021 (3)	0.003 (2)	−0.001 (2)	−0.005 (2)
C9	0.039 (3)	0.025 (3)	0.019 (3)	0.002 (2)	0.004 (2)	−0.002 (2)
C10	0.021 (2)	0.027 (2)	0.034 (3)	−0.0025 (18)	−0.001 (2)	0.000 (2)
C11	0.032 (3)	0.033 (3)	0.030 (3)	0.002 (2)	−0.001 (2)	0.001 (2)
C12	0.029 (2)	0.024 (2)	0.020 (2)	−0.0033 (18)	0.0030 (18)	−0.001 (2)
C13	0.036 (3)	0.032 (3)	0.026 (3)	−0.004 (2)	0.011 (2)	−0.006 (2)
C14	0.033 (3)	0.038 (3)	0.024 (2)	−0.004 (2)	0.0004 (18)	−0.003 (2)
C15	0.031 (3)	0.039 (3)	0.033 (3)	0.004 (2)	0.000 (2)	0.004 (2)
C16	0.045 (3)	0.034 (3)	0.029 (3)	0.009 (2)	0.006 (2)	0.001 (2)
C17	0.040 (3)	0.027 (3)	0.020 (3)	0.000 (2)	0.003 (2)	0.000 (2)
N	0.026 (2)	0.031 (2)	0.019 (2)	0.0027 (17)	−0.0002 (15)	−0.0011 (18)
O1	0.0286 (18)	0.0299 (19)	0.032 (2)	0.0042 (13)	−0.0022 (14)	0.0018 (15)
O2	0.0333 (19)	0.029 (2)	0.0276 (19)	0.0021 (14)	0.0027 (14)	0.0049 (16)
O3	0.036 (2)	0.0321 (18)	0.0238 (18)	0.0025 (14)	0.0029 (14)	−0.0057 (15)
S	0.0338 (6)	0.0398 (7)	0.0275 (7)	0.0061 (6)	−0.0047 (4)	0.0051 (6)

### Geometric parameters (Å, °)

**Table:**

C1—O1	1.338 (6)	C9—H9	0.9500
C1—N	1.377 (6)	C10—O2	1.201 (6)
C1—S	1.632 (5)	C10—N	1.390 (6)
C2—O1	1.458 (5)	C10—C11	1.525 (7)
C2—C3	1.535 (7)	C11—O3	1.416 (6)
C2—H2A	0.9900	C11—H11A	0.9900
C2—H2B	0.9900	C11—H11B	0.9900
C3—C4	1.501 (6)	C12—O3	1.364 (6)
C3—N	1.516 (6)	C12—C13	1.392 (7)
C3—H3	1.0000	C12—C17	1.404 (7)
C4—C9	1.394 (7)	C13—C14	1.388 (7)
C4—C5	1.408 (7)	C13—H13	0.9500
C5—C6	1.371 (6)	C14—C15	1.386 (8)
C5—H5	0.9500	C14—H14	0.9500
C6—C7	1.396 (7)	C15—C16	1.407 (8)
C6—H6	0.9500	C15—H15	0.9500
C7—C8	1.379 (8)	C16—C17	1.375 (7)
C7—H7	0.9500	C16—H16	0.9500
C8—C9	1.370 (7)	C17—H17	0.9500
C8—H8	0.9500		
			
O1—C1—N	109.7 (4)	O2—C10—N	119.3 (4)
O1—C1—S	122.1 (3)	O2—C10—C11	121.4 (4)
N—C1—S	128.1 (4)	N—C10—C11	119.0 (4)
O1—C2—C3	106.0 (4)	O3—C11—C10	110.2 (4)
O1—C2—H2A	110.5	O3—C11—H11A	109.6
C3—C2—H2A	110.5	C10—C11—H11A	109.6
O1—C2—H2B	110.5	O3—C11—H11B	109.6
C3—C2—H2B	110.5	C10—C11—H11B	109.6
H2A—C2—H2B	108.7	H11A—C11—H11B	108.1
C4—C3—N	110.1 (4)	O3—C12—C13	125.1 (4)
C4—C3—C2	116.7 (4)	O3—C12—C17	115.5 (4)
N—C3—C2	100.5 (3)	C13—C12—C17	119.4 (4)
C4—C3—H3	109.7	C14—C13—C12	119.8 (5)
N—C3—H3	109.7	C14—C13—H13	120.1
C2—C3—H3	109.7	C12—C13—H13	120.1
C9—C4—C5	118.6 (4)	C15—C14—C13	121.2 (5)
C9—C4—C3	121.0 (4)	C15—C14—H14	119.4
C5—C4—C3	120.3 (4)	C13—C14—H14	119.4
C6—C5—C4	120.0 (5)	C14—C15—C16	118.7 (5)
C6—C5—H5	120.0	C14—C15—H15	120.6
C4—C5—H5	120.0	C16—C15—H15	120.6
C5—C6—C7	120.6 (5)	C17—C16—C15	120.6 (5)
C5—C6—H6	119.7	C17—C16—H16	119.7
C7—C6—H6	119.7	C15—C16—H16	119.7
C8—C7—C6	119.2 (4)	C16—C17—C12	120.2 (5)
C8—C7—H7	120.4	C16—C17—H17	119.9
C6—C7—H7	120.4	C12—C17—H17	119.9
C9—C8—C7	120.8 (5)	C1—N—C10	130.8 (4)
C9—C8—H8	119.6	C1—N—C3	111.6 (4)
C7—C8—H8	119.6	C10—N—C3	116.2 (4)
C8—C9—C4	120.7 (5)	C1—O1—C2	111.7 (3)
C8—C9—H9	119.7	C12—O3—C11	118.5 (4)
C4—C9—H9	119.7		
			
O1—C2—C3—C4	−124.8 (4)	C15—C16—C17—C12	−1.0 (8)
O1—C2—C3—N	−5.8 (5)	O3—C12—C17—C16	−179.0 (5)
N—C3—C4—C9	119.5 (5)	C13—C12—C17—C16	2.0 (7)
C2—C3—C4—C9	−126.8 (5)	O1—C1—N—C10	−163.8 (4)
N—C3—C4—C5	−58.7 (6)	S—C1—N—C10	14.6 (7)
C2—C3—C4—C5	54.9 (6)	O1—C1—N—C3	1.3 (5)
C9—C4—C5—C6	−0.1 (7)	S—C1—N—C3	179.7 (4)
C3—C4—C5—C6	178.1 (4)	O2—C10—N—C1	151.5 (5)
C4—C5—C6—C7	1.0 (7)	C11—C10—N—C1	−34.2 (7)
C5—C6—C7—C8	−1.2 (7)	O2—C10—N—C3	−13.1 (6)
C6—C7—C8—C9	0.7 (7)	C11—C10—N—C3	161.2 (4)
C7—C8—C9—C4	0.1 (7)	C4—C3—N—C1	126.7 (4)
C5—C4—C9—C8	−0.4 (7)	C2—C3—N—C1	3.0 (5)
C3—C4—C9—C8	−178.7 (4)	C4—C3—N—C10	−65.8 (5)
O2—C10—C11—O3	8.6 (6)	C2—C3—N—C10	170.5 (4)
N—C10—C11—O3	−165.6 (4)	N—C1—O1—C2	−5.5 (5)
O3—C12—C13—C14	179.9 (4)	S—C1—O1—C2	175.9 (4)
C17—C12—C13—C14	−1.2 (7)	C3—C2—O1—C1	7.3 (5)
C12—C13—C14—C15	−0.5 (8)	C13—C12—O3—C11	−2.3 (7)
C13—C14—C15—C16	1.5 (8)	C17—C12—O3—C11	178.8 (4)
C14—C15—C16—C17	−0.7 (8)	C10—C11—O3—C12	69.4 (5)

### Hydrogen-bond geometry (Å, °)

**Table:**

Cg1 and Cg2 are the centroids of the C4–C9 and C12–C17 rings, respectively.

**Table:**

*D*—H···*A*	*D*—H	H···*A*	*D*···*A*	*D*—H···*A*
C5—H5···O2^i^	0.95	2.29	3.202 (6)	162
C9—H9···O3^ii^	0.95	2.56	3.350 (6)	140
C11—H11b···S^iii^	0.99	2.87	3.814 (5)	160
C17—H17···Cg1^iv^	0.95	2.99	3.703 (5)	133
C8—H8···Cg2^ii^	0.95	2.79	3.523 (6)	135

Symmetry codes: (i) *x*, *y*−1, *z*; (ii) *x*, *y*, *z*−1; (iii) −*x*+3/2, *y*+1/2, −*z*+2; (iv) *x*, *y*, *z*+1.
